# Dichloroacetate (DCA) as a potential metabolic-targeting therapy for cancer

**DOI:** 10.1038/sj.bjc.6604554

**Published:** 2008-09-02

**Authors:** E D Michelakis, L Webster, J R Mackey

**Affiliations:** 1 Department of Medicine, University of Alberta, Edmonton, Canada; 2 Department of Oncology, University of Alberta, Edmonton, Canada

**Keywords:** mitochondria, metabolism, apoptosis, potassium channels, positron emission tomography, glycolysis

## Abstract

The unique metabolism of most solid tumours (aerobic glycolysis, i.e., Warburg effect) is not only the basis of diagnosing cancer with metabolic imaging but might also be associated with the resistance to apoptosis that characterises cancer. The glycolytic phenotype in cancer appears to be the common denominator of diverse molecular abnormalities in cancer and may be associated with a (potentially reversible) suppression of mitochondrial function. The generic drug dichloroacetate is an orally available small molecule that, by inhibiting the pyruvate dehydrogenase kinase, increases the flux of pyruvate into the mitochondria, promoting glucose oxidation over glycolysis. This reverses the suppressed mitochondrial apoptosis in cancer and results in suppression of tumour growth *in vitro* and *in vivo*. Here, we review the scientific and clinical rationale supporting the rapid translation of this promising metabolic modulator in early-phase cancer clinical trials.

## A paradigm shift is needed in cancer therapeutics

Although some battles have been won since the declaration of the ‘war on cancer’ in 1971 in the United States, the war is ongoing. Despite enormous investments from industry and the public, oncology has an impressively poor success rate in the clinical development of effective investigational drugs; less than a third of that in cardiovascular or infectious diseases (Kamb *et al*, 2007). Drug development in oncology has typically focused on targets essential for the survival of all dividing cells, leading to narrow therapeutic windows. Non-essential targets offer more selectivity but little efficacy. It is extremely rare to find an essential target that is unique to cancer cells; the dependence of CML cells on Ableson kinase is only induced by a chromosomal translocation in the malignant clone, making the efficacy and selectivity of imatinib for CML an exception in cancer therapy (Kamb *et al*, 2007). The most important reason for the poor performance of cancer drugs is the remarkable heterogeneity and adaptability of cancer cells. The molecular characteristics of histologically identical cancers are often dissimilar and molecular heterogeneity frequently exists within a single tumour. The view that ‘there are many different types of cancers’ is increasingly shared by scientists and clinical oncologists. This has important implications, including the realisation that specific drugs have to be developed and tested for molecularly defined tumours and effects in one might not necessarily be relevant to another cancer.

The biggest challenge remains the selective induction of cell death (mainly apoptosis) in cancer but not normal cells. Pragmatically, an ideal anticancer therapy would be easily administered (possibly an orally available small molecule) and affordable. Most new anticancer drugs are prohibitively expensive not only for millions of patients from developing countries, but also for many patients without strong medical insurance in developed countries.

One way that the problem of heterogeneity of ‘proximal’ molecular pathways in cancer can be addressed is by targeting more ‘distal’ pathways that integrate several proximal signals, as long as the common distal pathways remain essential and specific to cancer cells. The unique metabolism of most solid tumours integrates many proximal pathways and results in a remodeling of mitochondria (where the regulation of energy production and apoptosis converge), to produce a glycolytic phenotype and a strong resistance to apoptosis. There is now growing evidence that the mitochondria might be primary targets in cancer therapeutics instead of simple bystanders during cancer development. This cancer-specific metabolic remodeling can be reversed by dichloroacetate (DCA), a mitochondria-targeting small molecule, that penetrates most tissues after oral administration (Bonnet *et al*, 2007; Pan and Mak, 2007). The molecular and direct metabolic response to DCA can also be followed by measuring glucose uptake in tumours by positron emission tomography (PET) imaging, non-invasively and prospectively. Such metabolic strategies might be able to shift the paradigm of experimental therapeutics in oncology.

The preclinical work on DCA (showing effectiveness in a variety of tumours and relatively low toxicity) (Bonnet *et al*, 2007), its structure (a very small molecule), the low price (it is a generic drug) and the fact that DCA has already been used in humans for more than 30 years, provide a strong rationale for rapid clinical translation. Here, we expand the scientific rationale and discuss several practical points that will be important in the clinical evaluation of DCA as anticancer therapy.

## The metabolism of cancer cells

**Figure 1:**
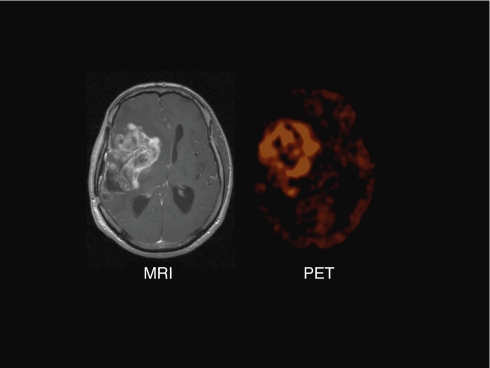
Brain MRI showing a large glioblastoma tumour with areas of necrosis within the tumour and significant brain oedema. On the right, a corresponding FDG-Glucose PET from the same patient shows much higher glucose uptake within the tumour, compared with the surrounding brain tissue.

Most cancers are characterised by aerobic glycolysis (GLY), that is, they use glycolysis for energy production, despite the fact that oxygen is present. In 1929, Warburg first observed this (i.e., the Warburg effect) and suggested it resulted from mitochondrial dysfunction, preventing the mitochondria-based glucose oxidation (GO) (Warburg, 1930). Because GO is far more efficient in generating ATP compared with GLY (producing 36 *vs* 2 ATP per glucose molecule), cancer cells upregulate glucose receptors and significantly increase glucose uptake in an attempt to ‘catch up’. Positron emission tomography imaging has now confirmed that most solid tumours have significantly increased glucose uptake and metabolism, compared with non-cancerous tissues (Figure 1). This bio-energetic difference between cancer and normal cells, might offer a very selective therapeutic target, as GLY is not typically seen in normal tissues apart from skeletal muscle during strenuous exercise. However, this area of experimental oncology has remained controversial; the glycolytic profile has traditionally been viewed as a result of cancer progression, not a cause and therefore the interest in targeting tumour metabolism has been low. Furthermore, at first glance, the glycolytic profile of cancer is difficult to understand, using an evolutionary model of carcinogenesis. First, why would these highly proliferating and energy-demanding cells rely on GLY rather than the much more efficient GO? Second, GLY results in significant lactic acidosis, which might cause significant toxicity to the surrounding tissues and the cancer cells themselves. Recent advances have caused a rekindling of the metabolic hypothesis of cancer suggesting that these facts are not as conflicting as they appear at first (Gatenby and Gillies, 2004):

## Glycolysis offers an early adaptation to the hypoxic microenvironment in carcinogenesis

Gatenby and Gillies (2004) recently proposed that as early carcinogenesis often occurs in a hypoxic microenvironment, the transformed cells have to rely on anaerobic GLY for energy production. Hypoxia-inducible factor (HIF) is activated in hypoxic conditions and it has been shown to induce the expression of several glucose transporters and most of the enzymes required for GLY (Semenza *et al*, 1994). For example, HIF induces the expression of pyruvate dehydrogenase kinase (PDK) (Kim *et al*, 2006), a gate-keeping enzyme that regulates the flux of carbohydrates (pyruvate) into the mitochondria. In the presence of activated PDK, pyruvate dehydrogenase (PDH) is inhibited, limiting the entry of pyruvate into the mitochondria, where GO can take place. In other words, activated PDK promotes completion of GLY in the cytoplasm with metabolism of pyruvate into lactate; inhibited PDK ensures an efficient coupling between GLY and GO.

Initially, tumours compensate by increasing glucose uptake into the cells. Furthermore, Gatenby and Gillies (2004) list a number of mechanisms through which lactic acidosis facilitates tumour growth: breakdown of extra-cellular matrix allowing expansion, increased cell mobility/metastatic potential and (along with HIF) activation of angiogenesis. Although tumours eventually become vascularised and are not significantly hypoxic anymore (although some tumours remain hypoxic at the core because the quality of the neo-vessel formation is poor) the aerobic glycolytic profile persists. This suggests that the (initially adaptive) metabolic remodeling confers a survival advantage to cancer cells. Indeed, recent evidence suggests that transformation to a glycolytic phenotype offers resistance to apoptosis (Plas and Thompson, 2002) (Kim and Dang, 2005, 2006).

## Glycolysis is associated with resistance to apoptosis

Several of the enzymes involved in glycolysis are also important regulators of apoptosis and gene transcription, suggesting that links between metabolic sensors, cell death and gene transcription are established directly through the enzymes that control metabolism (Kim and Dang, 2005). For example, hexokinase activation leads to a significant suppression of apoptosis; activated hexokinase translocates from the cytoplasm to the mitochondrial membranes where it interacts with and suppresses several key components of mitochondria-dependent apoptosis (Pastorino *et al*, 2005). It is therefore not surprising that hexokinase is upregulated and activated in many cancers (Kim and Dang, 2006). How does this occur? The promoter of hexokinase contains both p53 and HIF response elements and both mutated p53 and activated HIF increase hexokinase expression (Mathupala *et al*, 1997). In addition, the oncogenic protein Akt is upregulated in many cancers and induces a glycolytic metabolic profile through a number of mechanisms (Elstrom *et al*, 2004). Akt increases both the expression and activity of hexokinase (Gottlob *et al*, 2001; Elstrom *et al*, 2004). The gene that normally antagonises Akt, PTEN, is mutated (loss of function mutation) in a large number of cancers. Very recent data revealed even more links between p53 and metabolism: p53 regulates the expression of a critical enzyme of GLY through the production of TIGAR and is also directly regulating the expression of a subunit of *cytochrome c oxidase*, an important element of complex IV of the electron transport chain in mitochondria (reviewed in (Pan and Mak, 2007)). In other words, the most common molecular abnormality in cancer, that is, the loss of p53 function, induces metabolic and mitochondrial changes, compatible with the glycolytic phenotype. Likewise, the c-myc transcription factor increases the expression of many enzymes of GLY and can induce this same metabolic phenotype (Kim and Dang, 2005, 2006).

To conclude, an evolutionary theory of carcinogenesis identifies metabolism and GLY as a critical and early adaptive mechanism of cancer cells against hypoxia, that persist because it offers resistance to apoptosis in cancer cells (Gatenby and Gillies, 2004). The genetic theory on carcinogenesis, also identifies GLY and metabolism as an end result of activation of many diverse oncogenes, including c-myc, Akt/PTEN and p53 (Pan and Mak, 2007). Therefore, it is possible that this metabolic phenotype is centrally involved in the pathogenesis of cancer and is not simply a ‘by-product’ of carcinogenesis. Although it is not clear whether this metabolic phenotype directly induces malignancy, it certainly ‘facilitates’ carcinogenesis (Kim and Dang, 2006). In addition, this metabolic signature is the common denominator of multiple and diverse pathways; which means that if it is therapeutically targeted it might offer selectivity for malignant cells of diverse cellular and molecular origins.

## Mitochondria and apoptosis

**Figure 2:**
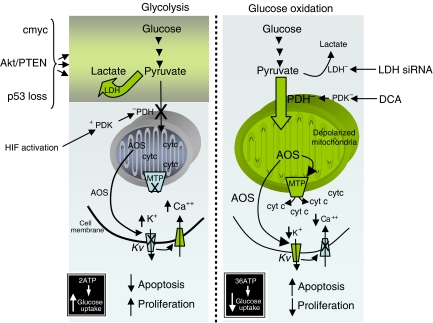
A glycolytic environment is associated with an antiapoptotic and pro-proliferative state, characterizing most solid tumours. Increase entry of pyruvate into the mitochondria by either DCA or inhibition of LDH, promotes glucose oxidation, increased apoptosis and decreased proliferation and tumour growth (see text for discussion).

Shifting metabolism away from mitochondria (GO) and towards the cytoplasm (GLY), might suppress apoptosis, a form of cell death that is dependent on mitochondrial energy production (Figure 2). Pro-apoptotic mediators, like cytochrome *c* and apoptosis-inducing factor, are protected inside the mitochondria. When the voltage- and redox-sensitive mitochondrial transition pore (MTP) opens, they are released in the cytoplasm and induce apoptosis, although it is possible that this can occur without MTP opening (Halestrap, 2005). Mitochondrial depolarisation and increased ROS are associated with opening of the MTP (Zamzami and Kroemer, 2001). Mitochondrial membrane potential and ROS production are dependent on the flux of electrons down the electron transport chain (ETC), which in turn are dependent on the production of electron donors (NADH, FADH_2_) from the Krebs’ cycle. Suppressing the entry of pyruvate into the mitochondria and thus the production of acetyl-CoA, will suppress both Krebs’ cycle and the ETC and thus MTP opening and apoptosis.

Mitochondria can also affect downstream mechanisms involved in proliferation and apoptosis. For example, mitochondria uptake can directly regulate intracellular Ca^++^, the increase of which is associated with increased proliferation and activation of many transcription factors. Also, the mitochondria-produced superoxide can be dismutated to H_2_O_2_ through the manganese superoxide dismutase and diffuse freely, activating plasma membrane K^+^ channels, thereby regulating the influx of Ca^++^ and the activity of caspases. K^+^ channels are transmembrane proteins allowing the passage of K^+^ ions through the plasma membrane. Closing of K^+^ channels or decreasing their expression results in an increase in [K^+^]_i_ which, in turn, increases the tonic inhibition that cytosolic K^+^ exerts on caspases (Remillard and Yuan, 2004). The voltage-gated family of K^+^ channels (Kv) is redox-sensitive and therefore can be regulated by the mitochondria. For example, mitochondria-derived H_2_O_2_ can activate certain Kv channels, like Kv1.5 (Bonnet *et al*, 2007).

## DCA reverses the mitochondrial remodeling, unlocking the cancer cells from a state of apoptosis resistance: preclinical work

We recently showed that several cancer cell lines (non-small cell lung cancer, breast cancer and glioblastoma) had hyperpolarised mitochondria, compared with non-cancer cell lines (Bonnet *et al*, 2007), a finding that was first described by Dr Chen at the Dana Farber Institute in the 1980s (Chen, 1988). This was associated with suppressed levels of mitochondria-derived ROS and decreased activity and expression of Kv channels. The Ca^++^-sensitive transcription factor NFAT was also active (i.e., nuclear) in the cancer cells. NFAT is a transcription factor that has been shown to increase the levels of the antiapoptotic bcl-2 and decrease the levels of the Kv channel Kv1.5. All of these features are compatible with an antiapoptotic state and could be secondary to a suppressed mitochondrial activity: decrease entry of pyruvate would eventually result in decrease flux of electrons in the ETC and therefore decreased ROS production, closing of the existing redox-sensitive Kv channels and increased intracellular Ca^++^. The decreased ROS could also contribute to closure of the redox-sensitive MTP and mitochondrial hyperpolarisation. The decreased entry of pyruvate into the mitochondria (and therefore the decreased GO) would result in compensatory GLY. Increased hexokinase levels would contribute to the hyperpolarisation of the mitochondria; increased hexokinase in a glycolytic environment is known to be translocated to the mitochondrial membrane, inhibiting the voltage-dependent anion channel (a component of the MTP), resulting in hyperpolarisation and suppression of apoptosis (Pastorino *et al*, 2005) (Figure 2).

**Figure 3:**
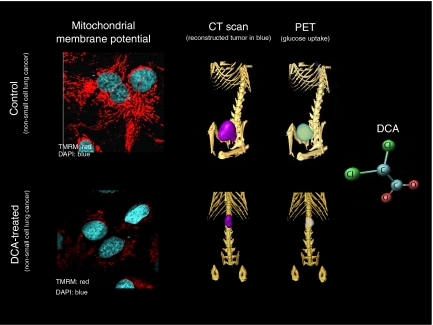
Dichloroacetate depolarises mitochondria and suppresses tumour growth *in vivo*. On the left, non-small cell lung cancer cells are loaded with TMRM before and after treatment with DCA (the higher the red fluorescence the higher the mitochondrial membrane potential; nuclei in blue). The same cells were injected in the flank of nude rats. On the right these rats are imaged with a rodent PET-CT (GammaMedica). Simultaneous CT and FDG-Glucose PET imaging shows that DCA therapy decreases both the size and the glucose uptake in the tumour.

Dichloroacetate activated the pyruvate dehydrogenase, which resulted in increased delivery of pyruvate into the mitochondria. As predicted, DCA increased GO and depolarised the mitochondria, returning the membrane potential towards the levels of the non-cancer cells, without affecting the mitochondria of non-cancerous cells (Figure 2). Remarkably, all the above features of the cancer cells were ‘normalised’ following the increase in GO and the mitochondrial depolarisation: ROS increased, NFAT was inactivated and function/expression of Kv channels was increased. Most importantly, apoptosis was induced in the cancer cells with both cytochrome *c* and apoptosis-inducing factor efflux from the mitochondria. This resulted in a decrease in tumour growth both *in vitro* and *in vivo* in xenotransplant models (Bonnet *et al*, 2007) (Figure 3). In addition to the induction of apoptosis by DCA in non-small cell lung cancer, breast cancer and glioblastoma cell lines reported in our original publication (Bonnet *et al*, 2007), very recently DCA was shown to induce apoptosis in endometrial (Wong *et al*, 2008) and prostate (Cao *et al*, 2008) cancer cells by largely the same mechanism, independently confirming our results. Furthemore, as predicted, activating mitochondria by DCA increases O_2_ consumption in the tumour and dramatically enhances the effectiveness of hypoxia-specific chemotherapies in animal models (Cairns *et al*, 2007).

It is important here to clarify that simply inhibiting GLY, will not promote pyruvate entry into the mitochondria, that is, it will not re-activate mitochondria. It will also be toxic to several non-cancerous tissues that depend on GLY for energy production. Inhibiting GLY (which has previously been tested as a potential treatment for cancer) results in ATP depletion and necrosis, not apoptosis, because apoptosis is an energy-consuming process, requiring active mitochondria (Xu *et al*, 2005). The ‘trick’ is to enhance the GLY to GO coupling, not just inhibit GLY. One of the ways that this can happen is by activating PDH, or inhibiting LDH, bringing pyruvate into the mitochondria and enhancing GO (Figure 2). This hypothesis is also supported by the recently published work that inhibition of LDH (by siRNA), which promotes the transfer of pyruvate into the mitochondria (in that sense mimicking DCA), also promotes cancer apoptosis and decreases tumour growth *in vitro* and in mice xenotransplants (Fantin *et al*, 2006).

## DCA: mechanism of action and clinical experience

Dichloroacetate is a small molecule of 150 Da (see structure in Figure 3) explaining in part the high bioavailability of this drug and the fact that it can penetrate into the traditional chemotherapy sanctuary sites, including the brain. *In vitro*, DCA activates PDH by inhibition of PDK at concentration of 10–250 *μ*M or 0.15–37.5 *μ*g ml^−1^ in a dose-dependent fashion (Stacpoole, 1989). To date, four different isoforms of PDK have been identified that have variable expression and sensitivity to the inhibition by DCA (Sugden and Holness, 2003). The isozyme constitutively expressed in most tissues and with the highest sensitivity to DCA is PDKII; in our published preclinical work we showed that PDK2 inhibition with siRNA completely mimicked DCA effects (Bonnet *et al*, 2007).

Oral DCA can achieve 100% bioavailability. Many studies using IV and oral DCA aimed to identify the optimal dose for DCA. The end point measured was the decrease in lactate levels in both the blood and the cerebrospinal fluid. A decrease in lactate levels is the immediate result of the inhibition of PDK (and thus activation of PDH) by DCA. Several studies treated patients with DCA and directly measured PDH activity in muscle biopsies. Dichloroacetate administered at 35–50 mg kg^−1^ decreases lactate levels by more than 60% and directly activates PDH by 3–6 fold (Howlett *et al*, 1999; Parolin *et al*, 2000).

Although the pharmacokinetics of DCA in healthy volunteers follow a simple one-compartment model, they are more complex in severely abnormal states like severe lactic acidosis or cirrhosis. Dichloroacetate inhibits its own metabolism by an unknown mechanism, and the clearance of DCA decreases after multiple doses (Stacpoole *et al*, 2003). Although the initial half-life with the first dose is less than one hour, this half-life increases to several hours with subsequent doses. However, there is a plateau of this effect and DCA serum levels do not continue to rise with chronic use. This is also true for DCA metabolites (which do not have any biologic effect, at least on PDH). For example, the serum DCA levels after 5 years of continued treatment with oral DCA at 25 mg kg^−1^ are only slightly increased compared with the levels after the first several doses (and remain in the range of approximately 100 *μ*g ml^−1^) (Mori *et al*, 2004). The effects on lactate levels are sustained and persist after the DCA levels decrease, because the inhibition of PDK is not immediately reversible; DCA ‘locks’ PDK in a sustained inactive state.

A large number of children and adults have been exposed to DCA over the past 40 years, including healthy volunteers and subjects with diverse disease states. Since its first description in 1969 (Stacpoole, 1969), DCA has been studied to alleviate the symptoms or the haemodynamic consequences of the lactic acidosis complicating severe malaria, sepsis, congestive heart failure, burns, cirrhosis, liver transplantation and congenital mitochondrial diseases. Single-arm and randomised trials of DCA used doses ranging from 12.5 to 100 mg kg^−1^ day^−1^ orally or intravenously (reviewed in (Stacpoole *et al*, 2003)). Although DCA was universally effective in lowering lactate levels, it did not alter the course of the primary disease (for example sepsis).

More than 40 nonrandomised trials of DCA in small cohorts of patients have been reported, but the first two randomised control trials of chronic oral therapy with DCA in congenital mitochondrial diseases were reported in 2006. In the first, a blinded placebo-controlled study was performed with oral DCA administered at 25 mg kg^−1^ day^−1^ in 30 patients with MELAS syndrome (mitochondrial myopathy, encephalopathy, lactic acidosis and stroke-like episodes) (Kaufmann *et al*, 2006). Most patients enrolled in the DCA arm developed symptomatic peripheral neuropathy, compared with 4 out of 15 in the placebo arm, leading to the termination of the study. Seventeen out of 19 patients had at least partial resolution of peripheral neurological symptoms by 9 months after discontinuation of DCA. This neurotoxicity resembled the pattern of length-dependent, axonal, sensorimotor polyneuropathy without demyelination. No other toxicities were reported. It is important to note that peripheral neuropathy often complicates MELAS because of primary or secondary effects on peripheral nerves; for example these patients also have diabetes and diabetes-related peripheral neuropathy.

In contrast, another randomised placebo-controlled double-blinded study failed to show any significant toxicity of DCA, including peripheral neuropathy. In this study only one of 21 children with congenital lactic acidosis treated with DCA orally at 25 mg kg^−1^ day^−1^ for 6 months demonstrated mild peripheral neuropathy. Serial nerve conduction studies failed to demonstrate any difference in incidence of neuropathy in the 2 arms (placebo *vs* DCA). Sleepiness and lethargy, muscular rigidity of the upper extremity and hand tremor were reported in one patient in each group (Stacpoole *et al*, 2006).

The higher incidence of peripheral neuropathy in adult MELAS patients may represent an intrinsic predisposition to this complication in MELAS or its associated conditions, that is, diabetes mellitus; this toxicity might also be age-dependent. In summary, peripheral neuropathy is a potential side effect of DCA that appears to be largely reversible. As peripheral neuropathy is a frequent complication of taxane, platinum and vinca-alkaloid chemotherapies, the risk for DCA-associated peripheral neuropathy may depend on whether cancer patients have prior or concurrent neurotoxic therapy.

## DCA: clinical testing in cancer?

There is substantial evidence in preclinical *in vitro* and *in vivo* models that DCA might be beneficial in human cancer (Bonnet *et al*, 2007; Cairns *et al*, 2007; Cao *et al*, 2008; Wong *et al*, 2008). The concept is strengthened by the fact that LDH inhibition in mice with human cancer xenotransplants, also induced apoptosis and inhibited growth, improving survival (Fantin *et al*, 2006). There is also 40 years of human experience with mechanistic studies of DCA in human tissues after oral use, pharmacokinetic and toxicity data from randomised studies for 6 months, and 5-year use case reports. This supports an easy translation to early-phase clinical trials.

Dichloroacetate could be tested in a variety of cancer types. The realisation that (i) a diverse group of signalling pathways and oncogenes result in resistance to apoptosis and a glycolytic phenotype, (ii) the majority of carcinomas have hyperpolarised/remodeled mitochondria, and (iii) most solid tumours have increased glucose uptake on PET imaging, suggest that DCA might be effective in a large number of diverse tumours. However, direct preclinical evidence of anticancer effects of DCA has been published only with non-small cell lung cancer, glioblastoma and breast, endometrial and prostate cancer. In addition, the lack of mitochondrial hyperpolarisation in certain types of cancer, including oat cell lung cancer, lymphomas, neuroblastomas and sarcomas (Chen, 1988), suggest that DCA might not be effective in such cases. Cancers with limited or no meaningful therapeutic options like recurrent glioblastoma or advanced lung cancer should be on top of the list of cancers to be studied.

No patient with cancer has received DCA within a clinical trial. It is unknown whether previously studied dose ranges will achieve cytotoxic intra-tumoral concentrations of DCA. In addition, the overall nutritional and metabolic profile of patients with advanced cancer differs from those in the published DCA studies. Furthermore, pre-exposure to neurotoxic chemotherapy may predispose to DCA neurotoxicity. Carefully performed phase I dose escalation and phase II trials with serial tissue biopsies are required to define the maximally tolerated, and biologically active dose. Clinical trials with DCA will need to carefully monitor neurotoxicity and establish clear dose-reduction strategies to manage toxicities. Furthermore, the pharmacokinetics in the cancer population will need to be defined.

The preclinical experience with DCA monotherapy warrants clinical trials with DCA as a single agent or in direct comparison with other agents. However, as it ‘unlocks’ cancer cells from a state of apoptosis resistance, DCA might be an attractive ‘apoptosis-sensitizer’ agent. In that sense, DCA could both precede and be given concurrently with chemotherapy or radiation therapy, in an attempt to increase their effectiveness, decrease the required doses and limit the toxicity of standard therapies (Cairns *et al*, 2007).

The ability to approach metabolism as an integrator of many diverse signalling pathways, prompts consideration of the imaging and diagnostic studies that might track metabolic modulation. As discussed above, important questions that need to be answered in clinical trials using DCA include: (i) can PET be used as a predictor of clinical response or as a means of documenting non-invasively a reversal of the glycolytic phenotype in response to DCA? (ii) can mitochondrial membrane potential or the acute effects on DCA in fresh tumour biopsies, predict clinical response to DCA and facilitate patient selection?

Funding for such trials would be a challenge for the academic community as DCA is a generic drug and early industry support might be limited. Fundraising from philanthropies might be possible to support early phase I–II or small phase III trials. However, if these trials suggest a favourable efficacy and toxicity, the public will be further motivated to directly fund these efforts and national cancer organisations like the NCI, might be inspired to directly contribute to the design and structure of larger trials. It is important to note that even if DCA does not prove to be the ‘dawn of a new era’ (Pan and Mak, 2007), initiation and completion of clinical trials with a generic compound will be a task of tremendous symbolic and practical significance. At this point the ‘dogma’ that trials of systemic anticancer therapy cannot happen without industry support, suppresses the potential of many promising drugs that might not be financially attractive for pharmaceutical manufacturers. In that sense, the clinical evaluation of DCA, in addition to its scientific rationale, will be by itself another paradigm shift.

## Note to proof

Since the acceptance of this review two important papers have confirmed the novel anticancer effects of DCA in prostate and endometrial cancers: Wong JY *et al*, Dichloroacetate induces apoptosis in endometrial cancer cells. *Gynecol Oncol* June 2008; **109**(3): 394–402 and Gao *et al*, Dichloroacetate (DCA) sensitizes both wild-type and over expressing Bcl-2 prostate cancer cells *in vitro* to radiation. *Prostate* 1 August 2008; **68**(11): 1223–1231.
